# Cotinine: Beyond that Expected, More than a Biomarker of Tobacco Consumption

**DOI:** 10.3389/fphar.2012.00173

**Published:** 2012-10-10

**Authors:** Valentina Echeverria Moran

**Affiliations:** ^1^Research and Development, Department of Veterans Affairs, Bay Pines VA Healthcare SystemBay Pines, FL, USA; ^2^Tampa VA Healthcare SystemTampa, FL, USA; ^3^Department of Molecular Medicine, University of South FloridaTampa, FL, USA

**Keywords:** cotinine, tobacco, fear, memory, nicotine

## Abstract

A greater incidence of tobacco consumption occurs among individuals with psychiatric conditions including post-traumatic stress disorder (PTSD), bipolar disorder, major depression, and schizophrenia, compared with the general population. Even when still controversial, it has been postulated that smoking is a form of self-medication that reduces psychiatric symptoms among individuals with these disorders. To better understand the component(s) of tobacco-inducing smoking behavior, greater attention has been directed toward nicotine. However, in recent years, new evidence has shown that cotinine, the main metabolite of nicotine, exhibits beneficial effects over psychiatric symptoms and may therefore promote smoking within this population. Some of the behavioral effects of cotinine compared to nicotine are discussed here. Cotinine, which accumulates in the body as a result of tobacco exposure, crosses the blood-brain barrier and has different pharmacological properties compared with nicotine. Cotinine has a longer plasma half-life than nicotine and showed no addictive or cardiovascular effects in humans. In addition, at the preclinical level, cotinine facilitated the extinction of fear memory and anxiety after fear conditioning, improved working memory in a mouse model of Alzheimer’s disease (AD) and in a monkey model of schizophrenia. Altogether, the new evidence suggests that the pharmacological and behavioral effects of cotinine may play a key role in promoting tobacco smoking in individuals that suffer from psychiatric conditions and represents a new potential therapeutic agent against psychiatric conditions such as AD and PTSD.

## Introduction

**Figure 1:**
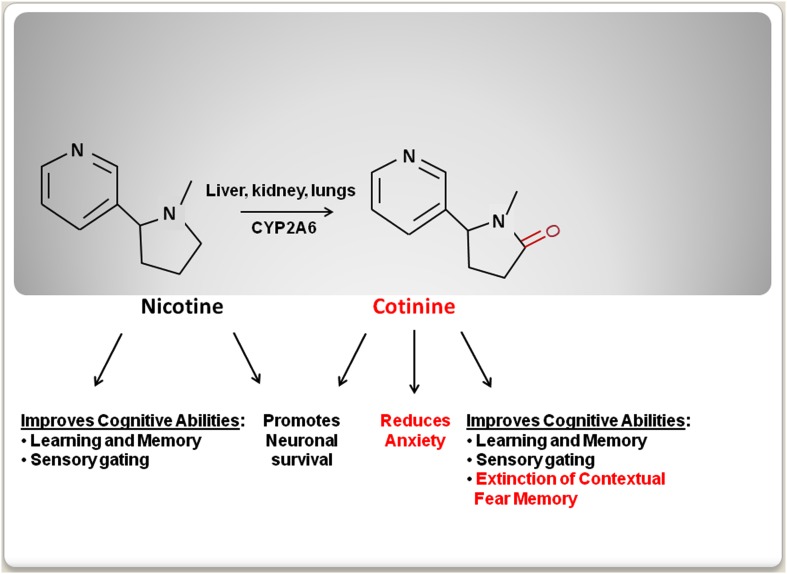
**A schematic comparison of the behavioral effects of nicotine and cotinine**.

The use of tobacco dates back as early as 5,000–3,000 BC, when tobacco plants were first cultivated in South America (Gately, 2003). Initially, tobacco was regarded has a medicinal plant to be used for medical purposes such as, a painkiller for earaches and toothaches (Balls, 1962). However, later studies clearly established the deleterious effects of tobacco smoking on health (Peto et al., 1996). As a result, the progressive establishment of more restrictive anti-tobacco laws has discouraged tobacco smoking worldwide. These new restrictions and public health campaigns have dramatically decreased tobacco use. However, there is a high rate of tobacco consumption among individuals that suffer from mental disorders such as, major depression disorder (MDD), schizophrenia, and post-traumatic stress disorder (PTSD; Leonard et al., 2001; Weaver and Etzel, 2003; Thorndike et al., 2006; Buggia-Prevot et al., 2008; Aubin et al., 2012). The idea that tobacco consumption in these populations is a form of self-medication is controversial and some evidence suggests that smoking is associated with poorer mental health outcomes in some mental disorders such as, bipolar and schizoaffective disorder (Dodd et al., 2010). The desire to identify the component of tobacco that may explain this correlation has encouraged the study of the mental effect(s) of nicotine [3-(1-methyl-2-pyrrolidinyl) pyridine], an alkaloid that is present in tobacco leaves, over the psychiatric symptoms. Nicotine treatment had been shown to improve cognitive function including attention, concentration, executive function, and learning and memory (Elzinga and Bremner, 2002; Horner and Hamner, 2002; Bowie and Harvey, 2005; Gray and Roth, 2007; Tapia et al., 2007; Burriss et al., 2008; Luck and Gold, 2008; Terry et al., 2008; Hinkelmann et al., 2009; Johnsen and Asbjornsen, 2009; Veltmeyer et al., 2009; Baune et al., 2010). However, nicotine’s undesirable cardiovascular and addictive side-effects have limited its therapeutic use (Karaconji, 2005). Recently, new studies have shown that the main metabolite of nicotine, an alkaloid named cotinine [(5*S*)-1-methyl-5-(3-pyridyl)-pyrrolidin-2-one], has beneficial therapeutic properties, while not having nicotine’s negative side-effects. In preclinical studies, cotinine has shown to improve reference and working memories, attention, and the extinction of fear memory, as well as to reduce both the startle response and anxiety in animal models of aging, Alzheimer’s disease (AD), PTSD, and schizophrenia (Figure 1). Here the psychopharmacology and the potential therapeutic use of cotinine are discussed.

## Discussion

### Cotinine pharmacokinetic

Cotinine is an alkaloid found in tobacco leaves and the main metabolite of nicotine. The active form of cotinine, the isomer S(-)-cotinine accumulates in the body after tobacco consumption. The pharmacokinetic profiles of cotinine administered orally or intravenously have been investigated in humans. These reports show that cotinine is well-absorbed orally (De Schepper et al., 1987), has a long plasma half-life (19–24 h; Benowitz et al., 1983; Benowitz and Sharp, 1989) and crosses the blood-brain barrier (Riah et al., 1998). Approximately 80–85% of nicotine is metabolized in the liver and converted into cotinine by enzymes such as cytochrome P450 2A6 (CYP2A6; Lewis et al., 1999) and cytochrome P4502A5 (CYP2A5) in human and mouse, respectively (Donato et al., 2000; Visoni et al., 2008).

In humans, cotinine is excreted in the urine, mainly as trans-3′-hydroxycotinine (90% of cotinine) and glucuronide (Caldwell et al., 1992; Ghosheh and Hawes, 2002; Kuehl and Murphy, 2003).

The expression of different variants of the CYP2A6 gene, coding for enzymes with different catalytic activities, influences the level of cotinine achieved in the body after nicotine consumption and, seems to influence nicotine addiction (Malaiyandi et al., 2006; Strasser et al., 2007). For example, individuals that express a catalytically deficient form of CYP2A6 (i.e., CYP2A6*4) showed a lower rate of cigarette consumption compared with individuals that express the normal allele (Yamanaka et al., 2004). The alleles that express the polymorphic forms of the CYP2A6 gene, with low or high enzymatic activity, are distinctly represented in diverse ethnic groups (Bramer and Kallungal, 2003; Nakajima et al., 2006). The distinct expression of the different forms of CYP2A6 may explain the varying metabolism of cotinine observed in individuals of diverse ethnic backgrounds (Nakajima et al., 2006). Therefore cotinine metabolism and, consequently, smoking behavior can be influenced by ethnicity. Furthermore, other factors such as the consumption of a specific food can also affect cotinine metabolism (Nakajima et al., 2006). For example, some components of grapefruit juice inhibit the activity of CYP2A6 and, consequently, cotinine synthesis (Tassaneeyakul et al., 2000; Hukkanen et al., 2006).

### Cotinine pharmacodynamic effects

Early studies of cotinine effects in humans showed that cotinine has a good safety profile (Borzelleca et al., 1962; Bowman and Mc, 1962). One of these seminal studies demonstrated that daily doses of cotinine of up to 1,800 mg for a period of 4 days did not induce deleterious side-effects in humans (Bowman and Mc, 1962). Another clinical study investigating the psychogenic effects of cotinine showed that when administered intravenously to abstinent smokers, this compound reduced the self-reported irritability and tobacco cravings experienced by the participants (Benowitz et al., 1983). A follow-up phase II clinical study investigated the effects of cotinine on smoking cessation in an inpatient, 10-day study in abstinent cigarette smokers (Hatsukami et al., 1997). This study showed that oral cotinine treatment of up to 160 mg/day had no addictive, cardiovascular (e.g., heart rate and blood pressure), or behavioral effects in individuals between 21 and 42 years of age (Hatsukami et al., 1997). A follow-up study from the same research group also found that cotinine at the doses studied did not help with tobacco cessation and antagonized the reduction of the withdrawal symptoms induced by a nicotine patch (Hatsukami et al., 1998). These results confirmed that orally administered cotinine exhibits behavioral effects in humans, likely by modulating the nAChR sensitivity to agonists; however, this concept requires further experimental validation.

#### Cotinine is a cognitive enhancer in a mouse model of Alzheimer’s Disease

Cotinine has been shown to prevent working and reference memory loss in a mouse model of AD (Echeverria et al., 2011). In this study, the ability of cotinine to prevent plaque development and memory loss in the Tg6799 mice (Ohno et al., 2006) was investigated. Two-month-old Tg and same age non-Tg (NT) mice were orally treated with vehicle (PBS) or 2.5 mg/kg of cotinine for 4.5 months via gavage and tested for working memory using the circular platform, the Radial Arm Water maze (RAWM), and cognitive interference tests. These behavioral study showed that cotinine prevented memory loss in Tg6799 mice and significantly decreased plaques burden in the cortex of the Tg6799 mice. Cotinine treatment also reduced the number and size of the amyloid plaques. The decrease in plaque load also correlated with a decrease in the levels of insoluble Aβ in the cortex of the cotinine-treated Tg mice when compared with the vehicle-treated Tg mice (Echeverria et al., 2011). This evidence suggest that cotinine may be a new therapeutic agent against this devastating condition (Echeverria and Zeitlin, 2012).

#### Cotinine reduced anxiety and fear in a mouse model of PTSD – like symptoms

Post-traumatic stress disorder is an anxiety disorder that appears after exposure to life-threatening events (Martenyi et al., 2007). PTSD is characterized by symptoms such as anxiety, fear, working memory impairment, hyperarousal, emotional numbing, and sleep disorders. These symptoms have been associated with a permanent alteration of the physiological response to stress induced by over-activation of the hypothalamus-pituitary-adrenal axis (Yehuda et al., 1991). These changes are generally accompanied by dysregulation of several neurotransmitter systems including the serotoninergic, dopaminergic, noradrenergic, and cholinergic systems. The hyperarousal and aggressive behavior observed in patients with PTSD are caused, at least in part, by a decrease in serotonin neurotransmission (Nutt, 2000). Consistent with this idea, serotonin reuptake inhibitors (SSRIs) such as paroxetine and sertraline are currently used in the treatment of PTSD (Brady et al., 2000; Davidson et al., 2001; Brady and Clary, 2003; Corchs et al., 2009; Stein et al., 2009). Because serotonin promotes acetylcholine release, and acetylcholine signaling positively affects attention and memory, an increase of serotonin levels in the brain, may simultaneously improve mood, reduce aggressiveness (Buhot et al., 2000), and positively affect cognitive abilities. Although SSRIs are useful in the treatment of PTSD (Cohen et al., 2000), they are effective only in a small percentage of patients (approximately 30%; Veltmeyer et al., 2009).

Post-traumatic stress disorder patients are commonly heavy smokers, and an association between tobacco dependence and PTSD has previously been established (Hapke et al., 2005). In the search of new drugs that could diminish fear and anxiety and enhance the extinction of fear memories, rodent models of fear conditioning (FC) has been extensively used. These animal models have permitted to characterize the effect of drugs, including cotinine, over anxiety, fear, and contextual memory in anxiety disorders. FC is a broadly used model of associative memory that involves the pairing of neutral conditioning stimuli (CS; sound and context) with an aversive unconditioned stimulus (US; electric shock). After conditioning, the presentation of the CS alone elicits both a conditioned fear response (freezing behavior) and anxiety in the animal. Experimentally, the extinction of contextual fear (FE) after FC is expressed as a progressive decrease in fear responses attained after repetitive exposures to the CS (context) in the absence of the pairing with an aversive US (Steckler and Risbrough, 2012).

A devastating symptom in PTSD is the failure to extinguish traumatic memories that force the patient to continue re-experiencing the trauma (Izquierdo et al., 2004). Thus, the identification of drugs that would enable the extinction of fear memories would be an essential therapeutic goal.

One recent study investigated the effect of pre- and post-treatment with cotinine over the stability of contextual fear memory after repetitive or single re-exposure to the CS (Zeitlin et al., 2012). Male adult mice were pre-treated or post-treated with cotinine before or after FC, respectively, and tested for fear responses and anxiety after being exposed to the CS (context). These studies showed that cotinine accelerated the extinction and reduced the stability of the contextual fear memory. FE is considered a “new learning” process that involves the acquisition of inhibitory memories that compete with the original fear memory consolidation. For this reason, it has been proposed that cognitive enhancers may have a positive effect over the extinction of fear memories. Thus the improvement of cognitive abilities induced by cotinine may be relevant for its effects on FE. Thus, cotinine may promote the extinction of contextual fear memory by stimulating the acquisition of new inhibitory memories.

In addition, because cotinine reduces anxiety and helps to extinguish fear memory, it is feasible that cotinine may also be useful, alone or in a combined treatment, with psychotherapy to reduce both non-cognitive and cognitive symptoms of PTSD. However, these ideas need to be tested in placebo-controlled clinical trials.

#### Cell signaling changes associated with fear extinction triggered by cotinine

This enhancement of contextual FE in mice subjected to FC described above, correlated with an increase in the extracellular signal-regulated kinases (ERK)1/2 (ERKs) activity in the hippocampus (Chen et al., 2005; Fischer et al., 2007). This increase has been regarded as a key molecular event during the fear extinction process because this brain region is involved in contextual FE (Bouton et al., 2006; Quirk and Mueller, 2008) and also because that ERK1/2 inhibition immediately after memory retrieval prevented the extinction of contextual memory (Chen et al., 2005). Consistent with these findings, the enhancement of FE induced by cotinine treatment in the C57BL/6, mice positively correlated with an increase in the levels of the active form of ERK1/2 (phospho-ERKs) in the hippocampus.

In addition this study showed that cotinine also reduced anxiety in the conditioned mice. Since it has been demonstrated that cotinine inhibited serotonin reuptake and increased its spontaneous release in rat brains (Fuxe et al., 1979), an increase in serotonin in the brain induced by cotinine may explain the reduction in anxiety after FC in the cotinine-treated mice. A similar effect may also explain the common perception of a “calming effect” experienced by tobacco smokers.

### Cotinine, is a memory enhancer in animal models of schizophrenia-like symptoms

#### Effect of smoking on psychiatric symptoms in schizophrenia

Schizophrenia is a mental disorder characterized by cognitive deficits and both positive and negative symptoms (Van Snellenberg, 2009). The positive symptoms include delusions, hallucinations, and difficulty in thought organization and oral expression. The negative symptoms include deficits in attention and motivation, social withdrawal, and emotional numbness (Van Snellenberg, 2009). Although the causes of schizophrenia are not well understood, abnormalities in the activity of several neurotransmitters such as, dopamine, acetylcholine, gamma-aminobutyric acid (GABA), and glutamate have been proposed (Lang et al., 2007). Since the development of clozapine, current drugs used to treat schizophrenia are mostly antagonists of the serotoninergic/dopaminergic signaling systems. These drugs have been effective in the management of positive psychotic symptoms; however, they do not effectively target the negative symptoms and cognitive deficits observed in schizophrenic patients. Thus, the identification of new drugs are needed to adequately treat these symptoms (Biedermann and Fleischhacker, 2011; Karam et al., 2012).

Patients with schizophrenia may often die prematurely due to health risk factors commonly associated with this condition including obesity and smoking behavior (Sagud et al., 2009; van Os and Kapur, 2009; von Hausswolff-Juhlin et al., 2009; Kelly et al., 2011).

A percentage of patients report, that smoking helps in decreasing psychiatric symptoms (Glynn and Sussman, 1990) which, become worse during tobacco withdrawal (Dalack and Meador-Woodruff, 1996). There are few and incomplete clinical data supporting the therapeutic effects of cotinine in psychiatric conditions, however, in one clinical study it was found that smoking high-nicotine cigarettes, compared to smoking de-nicotinized cigarettes, reduced negative symptoms without affecting positive symptoms (Smith et al., 2002). This effect is considered to be the result of the increase of dopamine levels in the nucleus accumbens and prefrontal cortex induced by nicotine (Corrigall and Coen, 1991). This increase in dopamine may stimulate active coping, improve attention, environmental engagement, and emotional responding, that are commonly absent in schizophrenic patients with prominent negative symptoms.

Cognitive impairment is one of the main challenges experienced by these patients. Growing evidence suggests that a deficit in cholinergic neurotransmission plays an important role in mediating cognitive deficits (Araki et al., 2002; Levin, 2002; Money et al., 2012). This evidence suggests that activation of the nicotinic receptors can be useful in treating some symptoms of schizophrenia particularly the cognitive deficits (Taly et al., 2009; Toyohara and Hashimoto, 2010).

#### The nicotinic receptors as targets of cotinine effects in schizophrenia

The nicotinic receptors are involved in mediating attention, sensory gating, and learning and memory (AhnAllen, 2012; Yakel, 2012). The most broadly expressed nicotinic receptor (90%) is formed by α4 and β2 subunits and binds nicotine with high affinity while, not binding α-bungarotoxin (McGehee and Role, 1995); this receptor is highly represented in the striatum and substantia nigra and, poorly expressed in the neocortex and hippocampus (Rubboli et al., 1994b).

No less relevant, although less expressed, is the α7 nicotinic acetylcholine receptor (α7 nAChR), a low affinity receptor for nicotine that is highly expressed in the midbrain, neocortex, thalamus, and hippocampus, with lower levels in the striatum (Sugaya et al., 1990; Freedman et al., 1993; Rubboli et al., 1994a,b). This receptor that has high affinity for α-bungarotoxin (McGehee and Role, 1995) and, plays a key role mediating several cognitive functions including attention, memory, executive function, and sensory gating (Woodruff-Pak and Gould, 2002; Leiser et al., 2009; AhnAllen, 2012).

A cholinergic deficit has been proposed as a factor leading to cognitive impairment in schizophrenia. Numerous studies have shown that expression of the α7 nAChR is decreased in the brains of patients with schizophrenia when compared to healthy controls (Breese et al., 2000; Leonard et al., 2000). This idea is supported by clinical evidence showing that the acetylcholinesterase inhibitor, galantamine, which increases the synaptic levels of acetylcholine, improved the cognitive abilities in patients with schizophrenia (Ago, 2010; Ago et al., 2011).

The high rate of smoking in individuals with schizophrenia, although not a evidence of causation, has permitted speculation of the beneficial effect of a tobacco-derivative, but not smoking itself (Dalack and Meador-Woodruff, 1999) on some schizophrenia symptoms such as working memory impairment (Leonard et al., 1998, 2001). For example, evidence has been reported suggesting that α7 nAChR expression is significantly lower in schizophrenic non-smokers than in control non-smokers (Mexal et al., 2010).

In a rat model of Schizophrenia-like symptoms, the administration of nicotine attenuated the working memory deficits induced by the dopamine antagonist dizocilpine (Levin et al., 1998; Ciamei et al., 2001). In patients with schizophrenia, nicotine administration via a nasal spray has shown to ameliorate deficits in working memory, attention, and anxiety (Smith et al., 2006; Buckley et al., 2007). In addition, other clinical studies have shown that chronic treatment with nicotine improved the cognitive abilities in schizophrenic patients treated with antipsychotics (McEvoy et al., 1999; Freedman et al., 2008).

Several modulators of nAChRs have been tested against schizophrenia. For example, a partial agonist of the α7 nAChR, DMXB-A (GTS-21) was investigated in a phase 2 clinical study investigating the therapeutic effects of this drug over the symptoms of schizophrenia. Although GTS-21 improved some negative symptoms, it did not ameliorate the cognitive deficits observed in schizophrenic patients (Olincy and Stevens, 2007; Freedman et al., 2008).

Despite cotinine acting as a weak agonist (100 times less potent than nicotine) of the nAChRs, in rats it reversed the cognitive deficits induced by antagonism of the glutamate receptor, *N*-methyl-d-aspartate receptor (NMDAR), with ketamine. In this rodent model cotinine improved sustained attention and attenuated behavioral alterations induced by ketamine (Terry et al., 2012).

This pro-cognitive effect is not limited to rodent models of this disorder as cotinine, at similar doses, also improved performance accuracy on the delayed matching to sample (DMTS) task in aged rhesus monkeys. The DMTS task assesses recognition memory for novel non-verbal patterns, and tests short-term visual memory. This test has 19 outcome measures, including latency to response, the number of correct choices selected, and the probability of an error after a correct or incorrect response. In this task, cotinine-treated monkeys performed significantly better than vehicle-treated controls (Terry et al., 2005), indicating that cotinine improved visual short-term memory. These results also suggest that cotinine is a general cognitive enhancer that is useful in different species and types of cognitive abilities.

But the positive effects of cotinine are not limited to cognitive abilities. A recent study tested the effect of acute subcutaneous (0.03–10 mg/kg) and chronic oral administration of cotinine on sustained attention and behavioral alterations in rats induced by the glutamate (NMDA) antagonist MK-801 (Terry et al., 2012). The effects of cotinine were assessed in a five-choice serial reaction timed task (5CSRTT), a test broadly used to measure visual attention and impulsivity in rats. The 5CSRTT is implemented in a specially designed operant chamber with multiple response locations (“nine-hole box”) using food reinforcers to maintain performance on baseline sessions (about 100 trials). The test gives the rat a brief 0.5 s visual stimulus, measuring the time, and accuracy of the animal’s reactions, as well as the errors made by the rat. The 5CSRTT is used for measuring various aspects of attentional control over performance with its main measures of accuracy, premature responding, correct response latencies, and latency to collect earned food pellets (Robbins, 2002; Robinson et al., 2009).

The results showed that acute treatment with cotinine diminished MK-801-induced impairments in accuracy and elevations in timeout responses while increasing the number of completed trials. Furthermore, chronic treatment with cotinine induced similar beneficial effects even when the difficulties of the task were increased. The authors concluded that cotinine was useful in improving sustained attention and, in decreasing the impulsive and compulsive behaviors that were related to the postulated glutamate receptor signaling dysfunction in schizophrenia (Terry et al., 2012).

#### Effect of cotinine on sensory gating in animal models of schizophrenia

A deficit in sensory gating is also a characteristic of schizophrenic patients as well as patients with other psychiatric conditions such as PTSD, borderline personality disorder, and bipolar disorder (Braff et al., 1992; Swerdlow et al., 2006). Individuals with this deficit cannot filter out irrelevant stimuli, as their brains are unable to inhibit its responsiveness to similar and repeated stimuli. The activation of cholinergic nicotinic receptors in the hippocampus is one of the main molecular mechanisms that control this inhibitory gating. Because the α7 nAChR is required for supporting sensory gating abilities (Freedman et al., 1994), a cognitive ability that is reduced in the patients with schizophrenia, this receptor is considered a potential therapeutic target for diminishing sensory gating deficit in this disorder (Olincy and Stevens, 2007).

The NMDAR antagonist, dizocilpine, or the dopamine agonist, apomorphine are used in rodents to induce memory and sensory gating deficits resembling those observed in persons with schizophrenia (Seillier and Giuffrida, 2009). For example, apomorphine is used to induce in rodents the deficit in prepulse inhibition (PPI) observed in schizophrenia. PPI is the decrease in the startle response to an auditory stimulus after repetitive exposure to the stimulus and a measure of sensory gating. The sensory gating deficit is expressed as an increase in the startle response (Geyer, 2006). To assess the effect of drugs on PPI animals are treated with vehicle, or the study drug, and are exposed to a weak acoustic stimulus (the prepulse); then, changes in the reflexive flinching response (startle) displayed as a result of a second stimulus of higher intensity (the pulse) is measured. Effective antipsychotic agents prevent the inhibition of PPI induced by psychogenic compounds such as apomorphine and dizolcipine. Previous studies have shown that nicotine blocks the apomorphine-induced disruption of the PPI of the acoustic startle in rats (Suemaru et al., 2004).

In fact, several studies have reported a positive effect of smoking on sensory gating and cognitive function in schizophrenia. In one study, the relationship between sensory gating and smoking levels was assessed by investigating the correlation between PPI and smoking behavior in schizophrenic and control patients (Rabin et al., 2009). The authors found that schizophrenic non-smokers have poorer PPI performances than schizophrenic smokers, whose levels did not differ from non-schizophrenic controls.

In addition to the observed effects of nicotine on sensory gating, other studies have demonstrated that nicotine also improved other schizophrenic symptoms (Lyon, 1999). These results support the view that smoking is a form of self-medication to alleviate psychotic symptoms and the unpleasant side-effects of antipsychotics (Matthews et al., 2011). Interestingly, cotinine, the long-lived metabolite of nicotine, also ameliorated the apomorphine-induced deficits in PPI of the acoustic startle response in rats (Risner et al., 1985; Terry et al., 2005). This result suggested that cotinine may have antipsychotic effects and underlie the beneficial effects of nicotine on attention and sensory gating, in addition to its predicted positive effect over working memory. In addition, as mentioned before, cotinine facilitates fear extinction a measure of executive function which has been found also impaired in people suffering from schizophrenia (Holt et al., 2009). Unfortunately, no clinical studies have been performed to investigate the effect of cotinine over these symptoms in schizophrenia.

### Molecular mechanisms potentially mediating the beneficial effects of cotinine on cognition

Thus far, the molecular mechanisms of cotinine action have been elusive. A previous report suggested that cotinine binds to an unknown type of receptor (Riah et al., 2000). Unfortunately, no follow-up studies pursued the characterization of this “cotinine receptor”. Other study based in the fact that cotinine increased serotonin levels in the rat brain, investigated whether granisetron, a 5HT(3) receptor antagonist, could enhance the efficacy of the nicotine patch. The results indicated that 5HT(3) antagonism was an unlikely mechanism of cotinine’s actions (Hatsukami et al., 2003). On the other hand, pharmacological evidence using ^125^I-labeled α-bungarotoxin and [^3^H] nicotine to differentiate subtypes of nAChRs affinities show that cotinine is a weak agonist of the α7 nAChRs and nicotine has more than 100 times higher affinity than cotinine for agonist binding sites of both subtypes of receptors. In addition the toxicological analysis using acute intraperitoneal injections of drugs in saline, showed that cotinine has in comparison to nicotine less than 1.5% toxicity (Table 1; Riah et al., 1999).

**Table 1:** **Potencies and affinities of cotinine and nicotine to different subtypes of nAChRs in the rat brain and values of acute toxicity in mice**.

	*I/IC_50_ [^3^H] nicotine	I/IC_50_^125^I-labeled α-bungarotoxin	Male		Female	
			LD_50_	LD_90_	LD_50_	LD_90_
Nicotine	100/(2 ± 0.1) × 10^−7^	100/(1 ± 0.3) × 10^−5^	31 ± 4	43 ± 6	37 ± 6	51 ± 9
Cotinine	100/(2 ± 0.2) × 10^−3^	100/(1 ± 0.2) × 10^−3^	2 ± 0.1	4 ± 0.1	3 ± 0.1	4 ± 0.1

*I/IC50, percentage maximum inhibition/concentration of drug that inhibits 50% radioligand binding (in M). Values of IC50, mean ± SD. LD, lethal dose with nicotine attributed 100% toxicity (Riah et al., 1999).

It has been suggested that α7 nAChRs is not the main target of cotinine (Riah et al., 1999) and other type of receptors for cotinine have been suggested (Riah et al., 2000). Also, using [^3^H]dopamine release assays and ligand-binding autoradiography in monkey striatum, it was concluded that cotinine functionally interacts with both α4β2 and α3α6β2 nAChR subtypes in the caudate with a IC_50_ for the inhibition of specific agonists in the micromolar range (O’Leary et al., 2008). In recent years, we and others have proposed that cotinine functions as a positive allosteric modulator of α7 nAChRs (Buccafusco et al., 2009; Zeitlin et al., 2012). Positive allosteric modulators, are compounds that facilitate endogenous neurotransmission without directly stimulating the target receptors (Bertrand and Gopalakrishnan, 2007; Faghih et al., 2007). For example the compound PNU-120596, acts as a positive allosteric modulator of nAChRs both *in vitro* and *in vivo* (see Young et al., 2008). PNU-120596 inhibited the ability of amphetamine to suppress auditory gating in rats, suggesting its potential for use in schizophrenia, characterized by auditory gating deficits. The effects of PNU-120596 *in vitro* were shown to be mediated by α7 nAChRs. As a positive allosteric modulator, PNU-120596 positively modulates nicotinic cholinergic neurotransmission mostly by preventing receptor desensitization. Because, cotinine has been shown to be a memory enhancer (Echeverria et al., 2011), and also inhibit sensory gating disruption it has been speculated that can have similar beneficial effects on memory in AD or schizophrenia (Hajos and Rogers, 2010). However this is highly speculative at this point and needs to be demonstrated.

Another hypothesis proposed to explain the beneficial effects of cotinine on cognition is the theory of desensitization of specific population of the α7nAChRs. This hypothesis speculates that cotinine desensitization of the α7 nAChRs expressed on inhibitory GABAergic neurons of the hippocampus, may result in the activation of excitatory glutamate receptors mediating the synaptic plasticity changes required for memory (Buccafusco et al., 2007, 2009). Functional assays of human α7 nAChR expressed in *Xenopus leavis* oocytes showed that cotinine acted as weak agonists at the human α7 nAChR (1% response at 1 mM) and inhibited the response to ACh with IC_50_ value of 175 μM (Briggs and McKenna, 1998). Although this hypothesis is intriguing, the doses required for inducing the receptor desensitization are higher than those showing pharmacological effects and direct evidence that cotinine may have this effect in the brain is still missing. The idea of a desensitization of the α7nAChR induced by cotinine is contradictory with the fact that in mice, the chronic treatment with cotinine induced the activation of the Akt/GSK3β signaling pathway, which is activated by the α7 nAChR, in both the hippocampus and cortex (Echeverria et al., 2011). In fact, cotinine prevented apomorphine-induced deficits in PPI of acoustic startle in rats (Buccafusco and Terry, 2003), a behavioral task that greatly depends on the activity of the α7 nAChR. The effects of cotinine can be better explained by cotinine functioning as a positive allosteric modulator of the human α7 nAChR.

As a positive modulator, cotinine may improve learning and memory performance and reverse the apomorphine-induced deficits of PPI, in addition to stimulating the protein kinase B (Akt)/GSK3β pathway. Furthermore, stimulation of α7 nAChR signaling may explain the neuroprotective effects of cotinine because Akt can promote neuronal survival via several mechanisms including stimulation of the expression of anti-apoptotic factors such as CREB and Bcl-2 while inactivating pro-apoptotic enzyme such as Ask1 (Kim et al., 2001). Evidence of an allosteric effect of cotinine on the α7 nAChR or other receptors.

## Conclusion

Altogether the evidence suggests that cotinine is less toxic and has different mechanism(s) of action than nicotine. Cotinine’s properties and the preclinical evidence of its nootropic effects in animal models of psychiatric conditions, suggests that cotinine as a pure agent, in absence of nicotine, represents a new therapeutic agent to reduce anxiety, facilitate the extinction of fear memories, and improve attention and working memory in individuals with psychiatric conditions such as AD.

## Conflict of Interest Statement

The author declares that the research was conducted in the absence of any commercial or financial relationships that could be construed as a potential conflict of interest.
